# Laminated Hybrid Junction of Sulfur‐Doped TiO_2_ and a Carbon Substrate Derived from Ti_3_C_2_ MXenes: Toward Highly Visible Light‐Driven Photocatalytic Hydrogen Evolution

**DOI:** 10.1002/advs.201700870

**Published:** 2018-03-30

**Authors:** Wenyu Yuan, Laifei Cheng, Yurong An, Shilin Lv, Heng Wu, Xiaoli Fan, Yani Zhang, Xiaohui Guo, Junwang Tang

**Affiliations:** ^1^ Science and Technology on Thermostructural Composite Materials Laboratory Northwestern Polytechnical University 710072 Xi'an China; ^2^ State Key Laboratory of Solidification Processing Northwestern Polytechnical University 710072 Xi'an China; ^3^ Key Lab of Synthetic and Natural Functional Molecule Chemistry of Ministry of Education College of Chemistry and Materials Science Northwest University 710069 Xi'an China; ^4^ Department of Chemical Engineering University College London Torrington Place London WC1E 7JE UK

**Keywords:** carbon, doping, MXenes, photocatalytic materials, TiO_2_

## Abstract

TiO_2_ is an ideal photocatalyst candidate except for its large bandgap and fast charge recombination. A novel laminated junction composed of defect‐controlled and sulfur‐doped TiO_2_ with carbon substrate (LDC‐S‐TiO_2_/C) is synthesized using the 2D transition metal carbides (MXenes) as a template to enhance light absorption and improve charge separation. The prepared LDC‐S‐TiO_2_/C catalyst delivers a high photocatalytic H_2_ evolution rate of 333 µmol g^−1^ h^−1^ with a high apparent quantum yield of 7.36% at 400 nm and it is also active even at 600 nm, resulting into a 48 time activity compared with L‐TiO_2_/C under visible light irradiation. Further theoretical modeling calculation indicates that such novel approach also reduces activation energy of hydrogen production apart from broadening the absorption wavelength, facilitating charge separation, and creating a large surface area substrate. This synergic effect can also be applied to other photocatalysts' modification. The study provides a novel approach for synthesis defective metal oxides based hybrids and broaden the applications of MXene family.

## 1 Introduction

A sustainable society heavily relies on clean and abundant energy supply.^1^ Hydrogen generation through the splitting of water by photocatalysis has been considered as a promising solution to the current energy and environmental dilemma.^2^ In the past decades, various semiconductors, such as TiO_2_,^3^ CdS,^4^ ZnO,^5^ C_3_N_4_,^6^ and WO_3_,^7^ were used to water splitting by harnessing solar energy.^8^ Among these materials, TiO_2_, a kind of semiconductor with a band gap of 3.0–3.2 eV, has been considered as one of promising candidates for photocatalytic H_2_ generation owing to its excellent stability and low cost.^9^

However, two main challenges remain for TiO_2_ photocatalysis: (i) rapid recombination of electrons–holes and (ii) low visible light absorption range owing to its large band‐gap energy.^10^ To improve the charge‐carrier separation efficiency, several strategies have been proposed, such as the introduction of noble metals,^11^ noble metal oxides, other semiconductors,^12^ and novel 2D nanomaterials.^13^ Particularly, the composite junction of TiO_2_ and highly conductive graphene was proved as promising photocatalysts owing to their abundance, large specific surface area (SSA), and low cost,^14^ more importantly charge separation due to the junction structure, leading to enhanced hydrogen evolution activity.^15^ However, the other drawback of TiO_2_ photocatalyst still remains. On the other hand, the photocatalytic hydrogen evolution reaction (HER) is very similar to that in electrocatalytic HER, the material design principles in electrocatalytic fields can be applied when studying the effects of substrate on the reaction mechanism in photocatalysis. It is widely known that graphene structure usually deliver poor catalytic performance owing to the insufficient active sites in electrocatalytic fields.^16^ Previous studies on electrocatalysis suggested that some defects on catalysts could provide more active sites for catalytic reaction due to reduced activation energy or Gibbs free energy for H* (∆*G*_H*_), leading to enhanced hydrogen evolution performance.^17^

To improve the visible light absorption of TiO_2_, nonmetal doping (C, N, S) and metal doping (Fe, Cu, Ni) are used to be applied to decrease the band‐gap energy.^18^ For example, Mao and co‐workers first reported the photocatalytic activity of black TiO_2_ treated by H_2_ and found that the absorption wavelength was greatly improved.^19^ Recently, sulfur has been proved as a potential dopant which can significantly improve the visible light response owing to the similar electron structure with O atoms.^20^ The enhanced activity was also proved by theoretical calculation.^21^ However, doping is usually accompanied by defects, which can serve as carrier recombination centers and reduce the separation efficiency,^22^ while also act as reaction sites as mentioned above, so a balance between the two functions is crucial. Many studies recently focused on developing a doped TiO_2_ on graphene substrate, such as reduce Graphene oxide (rGO)/Cu‐TiO_2_,^23^ N‐TiO_2_/rGO,^24^ and Fe^3+^‐TiO_2_/rGO,^25^ which can favor electrons transfer from doped TiO_2_ to graphene thus facilitating carrier separation and realize a narrow band‐gap photocatalyst. However, as mentioned above, graphene does not facilitate proton reduction reaction.

Based on the discussion above, a sulfur‐doped TiO_2_ on carbon substrate was targeted in the study, which to the best of our knowledge, has seldom been reported nowadays. Furthermore, a laminated layer structure is always regarded to have large SSA, beneficial to surface chemical reactions. To synthesize a layered photocatalyst, 2D transition metal carbides—*MXenes*, which are derived from the selectively etching of “*A*”‐layers from “*MAX*” phases, where M is a transition metal, A is A‐group element (Al or Si), and X is carbon/nitrogen, were chosen as precursors.^26^ The abundant functional groups on the surface of MXenes make them can be easily doped by heteroatoms. Thus we applied a novel approach to synthesize laminated defect‐controlled carbon supported sulfur‐doped TiO_2_ junction photocatalyst (LDC‐S‐TiO_2_/C) involving a sulfur impregnation process of Ti_3_C_2_ *MXenes* and subsequent oxidation cascade process. The LDC‐S‐TiO_2_/C delivered a very high photocatalytic H_2_ evolution rate under visible light irradiation. A wide operation wavelength window to produce H_2_ was also achieved. The reason behind such novel activity was discussed and this interesting finding should shed a new insight in synthesis of high active laminated hybrid photocatalysts for energy conversion.

## 2 Results and Discussion

**Figure 1:**
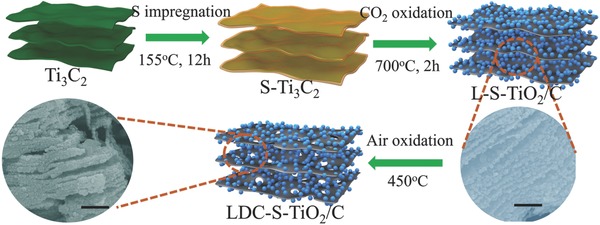
The schematic diagram of synthesis of LDC‐S‐TiO_2_/C. All scale bars are 500 nm.

The synthetic procedures for the LDC‐S‐TiO_2_/C are illustrated in **Figure** **1**. First, 2D Ti_3_C_2_ *MXenes* were synthesized via the selectively etching of Ti_3_AlC_2_ *MAX* phases. The morphology of Ti_3_C_2_ is shown in Figure S1 in the Supporting Information. A laminated structure can be obtained after selectively etching. After exfoliation, the d‐spacing of (002) plane was enlarged to 1.03 nm. Wherein, sulfur reactant was impregnated and adhered on the surface of Ti_3_C_2_ *MXenes* by a melt‐diffusion process. Then, the L‐S‐TiO_2_/C hybrids were fabricated via the mild CO_2_ oxidation of S‐Ti_3_C_2_ mixture. Finally, the LDC‐S‐TiO_2_/C hybrids were synthesized by the air oxidation of L‐S‐TiO_2_/C hybrids. As shown in Figure 1, the TiO_2_ nanoparticles with uniform particle size are anchored on ultrathin carbon layers. The structure is stable during air oxidation process because the structure has not changed obviously (Figure S2, Supporting Information). The average size of TiO_2_ nanoparticles (NPs) is ≈50 nm. However, the excess generated carbon in L‐S‐TiO_2_/C hybrids can shield light at the surface of TiO_2_.^27^ To reduce the amounts of carbon and increase defect concentration, the further oxidation process in air atmosphere was carried out. After the air oxidation, more TiO_2_ nanoparticles have been exposed.

**Figure 2:**
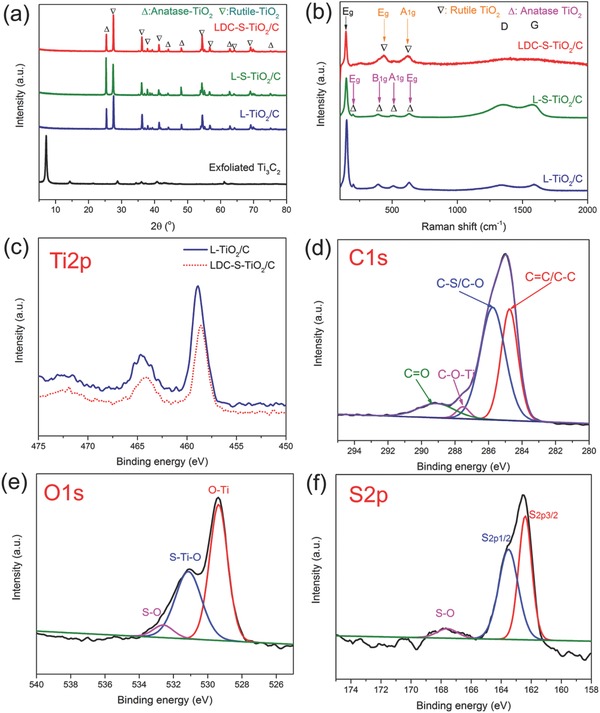
a) XRD patterns of exfoliated Ti_3_C_2_, L‐TiO_2_/C, L‐S‐TiO_2_/C, and LDC‐S‐TiO_2_/C. b) Raman spectra of L‐TiO_2_/C, L‐S‐TiO_2_/C, and LDC‐S‐TiO_2_/C. The Ti2p XPS c), C1S XPS d), O1s XPS e), and S2p XPS spectrum f) of LDC‐S‐TiO_2_/C. The Ti2p XPS of L‐TiO_2_/C was used as a reference sample to show the effects of sulfur doping on Ti2p XPS in (c).

The X‐ray diffraction (XRD) patterns are shown in **Figure** **2**a. After mild CO_2_ oxidation, the obvious diffraction peaks of two phase‐TiO_2_ can be observed in the XRD, meanwhile the diffraction peaks of Ti_3_C_2_ were disappeared, indicating the oxidation of Ti_3_C_2_ and the generation of anatase and rutile TiO_2_. With sulfur doping, the structure do not occur obvious change. However, the enlarged peaks between at 25°–30° treated in different oxidation temperature and CO_2_ flux show and both the diffraction peaks of anatase and rutile TiO_2_ undergo slight shift, as shown in Figure S3a,b in the Supporting Information. The phenomena that the diffraction peaks shift to lower 2θ degree indicate that the d‐spacing of TiO_2_ has been enlarged after sulfur doping.^28^ Furthermore, the largest d‐spacing change occurs at the condition of 700 °C with 150 sccm CO_2_, meanwhile the least occurs at the condition of 800 °C with 150 sccm of CO_2_. Therefore, the oxidation temperature was set at 700 °C and the gas flux was chosen at 150 sccm for later studies as maximum sulfur was doped under this condition. The air oxidation temperature was set at 450 °C because the oxidation of carbon began to happen under a temperature higher than 400 °C and high temperature (>500 °C) would lead to the unexpected rapid oxidation (Figure S4, Supporting Information). Besides, the diffraction peaks of rutile TiO_2_ in LDC‐S‐TiO_2_/C have been enhanced compared with L‐S‐TiO_2_/C, suggesting that more rutile TiO_2_ were produced during air oxidation process, which is consistent with the previous literature's conclusion.^29^ The ratio of rutile TiO_2_ to anatase TiO_2_, which is qualitatively calculated from XRD patterns, is about 4:1, indicating that rutile TiO_2_ is the main phase in LDC‐S‐TiO_2_ prepared at high temperature. To further identify the structure of carbon substrate and doped TiO_2_, the Raman spectra were carried out and the results are shown in Figure 2b. The Raman peaks at 1590 cm^−1^ (G band) and 1350 cm^−1^ (D band) indicate the existence of carbon, in which D band indicates the defects and G band resulted from the in‐plane vibration of sp^2^ carbon atoms. The LDC‐S‐TiO_2_/C presents weaker D band and G band than L‐S‐TiO_2_/C, which means that part of carbon has been oxidized during the second oxidization process, which would create more pores, increasing surface area. The *I*_D_/*I*_G_ of L‐TiO_2_/C, L‐S‐TiO_2_/C, and LDC‐S‐TiO_2_/C are 0.89, 1.04, and 1.18, respectively, suggesting that more functional groups or species are generated after sulfur doping and oxidation. The peaks of 196, 396, and 443 cm^−1^ can be assigned to be the rutile TiO_2_. With sulfur doping, the peaks of TiO_2_ have been weakened and broadened owing to the existence of defects.

To further explore the chemical bonds of Ti_3_C_2_ and LDC‐S‐TiO_2_/C, X‐ray photoelectron spectroscopy (XPS) was conducted in Figure S5 in the Supporting Information and Figure 2c–f. We first compared the Ti2p and C1s XPS spectra of Ti_3_C_2_ precursors and obtained LDC‐S‐TiO_2_/C to identify the complete transformation of precursors. As shown in Figure S5a,c in the Supporting Information, the Ti2p3/2 (454.3 eV) and Ti2p1/2 (460.3 eV) of Ti—C bonds could be clearly observed in Ti_3_C_2_ precursors. However, in LDC‐S‐TiO_2_/C, these typical bonds for Ti_3_C_2_ disappeared and the typical Ti2p 3/2 (458.6 eV) and Ti2p1/2 (464.3 eV) of TiO_2_ were generated. Besides, an obvious C—Ti bond (281.9 eV) in Ti_3_C_2_ also disappeared, suggesting that the Ti—C bond has been destroyed. Combining with the XRD and Raman spectra, we came to a conclusion that the Ti_3_C_2_ precursors have been totally oxidized and transformed into carbon and TiO_2_. Then the XPS was carried out to analyze the chemical bonds after sulfur doping. The XPS survey of LDC‐S‐TiO_2_/C is shown in Figure S6 in the Supporting Information and the results show that the elements are mainly composed of C, O, Ti, and S. The Ti 2p3/2 and Ti 2p1/2 are located at 458.6 and 464.3 eV, respectively (Figure 2c), which have slightly shifted (0.3 eV) to lower energy after S doping owing to the less electronegative.^30^ Four main peaks at 284.7, 285.8, 287.6, and 289.2 eV for C1s are assigned to C=C/C—C, C—O/C—S, C—O—Ti, and C=O bonds, respectively. The C—O/C—S and C=O bonds indicate that many defects existed in carbon layers. The O 1s peaks at 529.5, 531.2, and 532.5 eV can be assigned to Ti—O, S—Ti—O, and S—O bonds, respectively, suggesting that S replaced partial O atom in TiO_2_.^31^ Besides, we noticed that the peak of Ti—O bond has also shifted to lower binding energy. The S2p peaks at 162.0, 163.7, and 167.6 eV can be assigned to S2p3/2, S2p1/2, and S—O bonds, respectively (Figure 2f). The XPS spectra of L‐S‐TiO_2_ without carbon via 4 h oxidation of L‐S‐TiO_2_/C was shown in Figure S7 in the Supporting Information to further confirm that the sulfur atom was doped into TiO_2_ molecule not in the interfaces between carbon and TiO_2_. Compared to the XPS spectra of LDC‐S‐TiO_2_/C, the peak shift in Ti2p XPS spectra, the S—Ti—O bond and S—O bond in O1s XPS spectra still existed, indicating that sulfur atoms have been successfully doped into TiO_2_ molecules. Thus, we can confirm that S^2−^ replaces the O^2−^, achieving successfully synthesis of carbon supported S‐TiO_2_, in which the calculated percentage of sulfur is ≈3.4 at% via an elemental analyzer.

**Figure 3:**
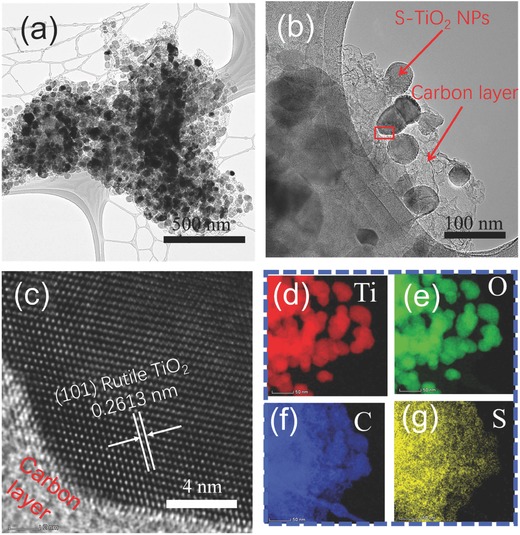
a) TEM images of LDC‐S‐TiO_2_/C. b,c) HRTEM of LDC‐S‐TiO_2_/C. d–g) Energy dispersive X‐ray (EDX) mapping of Ti, O, C, and S elements.

The transmission electron microscope (TEM) was carried out to show the architecture of the final sample LDC‐S‐TiO_2_/C. The TiO_2_ NPs can be clearly observed in **Figure** **3**a. The TiO_2_ NPs with average particle size of ≈50 nm and the carbon layers can be further identified from the high‐resolution TEM (HRTEM) (Figure 3b). The total structure has been well preserved during air oxidation process, as shown in Figure S2 in the Supporting Information. Furthermore, owing to that the ionic radius of S^2−^ (0.184 nm) is larger than that of O^2−^ (0.136 nm),^32^ the d‐spacing of 0.2613 nm (Figure 3c) is corresponding to the (101) plane of rutile TiO_2_, which is larger than that of nondoped rutile TiO_2_ (Figure S8, Supporting Information). The sulfur element can be clearly observed in the mapping analysis of L‐S‐TiO_2_ sample without carbon (Figure S9, Supporting Information) indicated that sulfur atoms have been successfully doped into TiO_2_ molecules. Further elemental analysis confirms that O, C, Ti, and S are homogeneously distributed on LDC‐S‐TiO_2_/C, as shown in Figure 3d–g, and the corresponding image is shown in Figure S10 in the Supporting Information.

The SSA and pore structure was measured by Brunauer–Emmett–Teller (BET) method and the results are shown in Figure S11 in the Supporting Information. With S doping, the SSA of L‐S‐TiO_2_/C reaches 57.2 m^2^ g^−1^ owing to more micropores were introduced by doping, compared to that of L‐TiO_2_/C is only 45 m^2^ g^−1^. The improved SSA can contribute more active sites caused by S doping. Furthermore, after oxygen oxidation, the SSA of LDC‐S‐TiO_2_/C reaches up to 79.9 m^2^ g^−1^. The pore size distribution (PSD) in Figure S11b in the Supporting Information suggests that the LDC‐S‐TiO_2_/C exhibits abundant pore structure. The enhanced SSA and PSD indicate that the LDC‐S‐TiO_2_/C can expose more S‐doped TiO_2_ for the light absorption and favor the hydrogen evolution reaction compared with L‐TiO_2_/C and L‐S‐TiO_2_/C, leading to enhanced photocatalytic activity.

**Figure 4:**
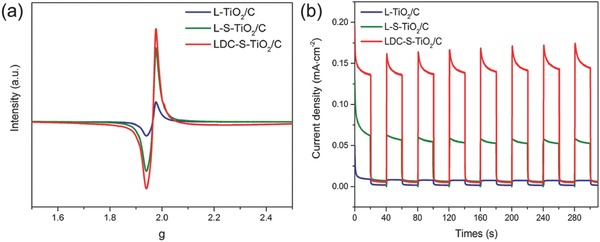
a) Electron paramagnetic resonance (EPR) spectra of L‐TiO_2_/C, L‐S‐TiO_2_/C, and LDC‐S‐TiO_2_/C. b) Transient photocurrent density (TPC) response of L‐TiO_2_/C, L‐S‐TiO_2_/C, and LDC‐S‐TiO_2_/C.

The oxygen vacancies were investigated by using electron paramagnetic resonance (EPR) spectrometer. From the EPR spectra in **Figure** **4**a, it is observed that there is a main resonance line in the spectra located at the electron's g‐factor of around 1.96, which is attributed to the unpaired electrons trapped on oxygen vacancies (singly ionized oxygen vacancy *V*_O_^•^). L‐S‐TiO_2_/C shows higher *V*_O_^•^ concentration than L‐TiO_2_/C owing to the sulfur doping. More interestingly, the EPR intensity of LDC‐S‐TiO_2_/C is further slightly improved. Considering that the carbon can be easily oxidized by O_2_, the enhanced *V*_O_^•^ concentration is mainly attributed to the oxidation of carbon, which is consistent with the Raman spectra.^33^ Furthermore, the charge‐transfer efficiency was investigated using electrochemical impedance spectroscopy (EIS) and transient photocurrent density (TPC) response measurements, respectively. As indicated in Figure S12 in the Supporting Information, L‐S‐TiO_2_/C shows a much smaller semicircle diameter and a much lower interfacial charge‐transfer resistance than those of L‐TiO_2_/C in potassium phosphate buffer solution (pH = 7) under visible‐light irradiation, suggesting the apparent enhancement of interfacial charge‐carrier transfer on the surface of L‐S‐TiO_2_/C. After further oxidation, the charge‐transfer resistance of LDC‐S‐TiO_2_/C is further reduced owing to the improved charge transfer. The TPC response results in Figure 4b shows that the highest photoinduced current density is achieved by LDC‐S‐TiO_2_/C, reaching 0.135 mA cm^−2^, about two times that of L‐S‐TiO_2_/C (0.062 mA cm^−2^), and about nine times that of L‐TiO_2_/C (0.014 mA cm^−2^). Hence, sulfur doping greatly enhances the absorption wavelength region and forms a junction with carbon substrate that significantly improves the charge separation efficiency, leading to high photoinduced current density.

To investigate the light absorption ability of LDC‐S‐TiO_2_/C, the UV–vis spectra were measured as shown in Figure S13 in the Supporting Information. The undoped LDC‐TiO_2_/C was taken as a reference sample. The UV–vis spectra of L‐S‐TiO_2_/C and L‐TiO_2_/C are not included because excess carbon makes the absorbance higher than 1 and the band‐structure unclear. Therefore, we mainly compare the spectra of LDC‐S‐TiO_2_/C and LDC‐TiO_2_/C. The nondoped LDC‐TiO_2_/C displays the maximum absorbance at a wavelength of 400 nm with a threshold wavelength of 430 nm (*E*_g_ = 2.88 eV). After S doping, LDC‐S‐TiO_2_/C shows enhanced absorption intensity in visible wavelength and displays two band gaps. One is similar to nondoped LDC‐TiO_2_/C owing to the intrinsic band gap of TiO_2_. The other is calculated as 1.62 eV, indicating that the near‐infrared visible light can be utilized by LDC‐S‐TiO_2_/C. The observed shift of the absorption indicates that sulfur doping is effective in extending the optical response of TiO_2_ in visible wavelength.

**Figure 5:**
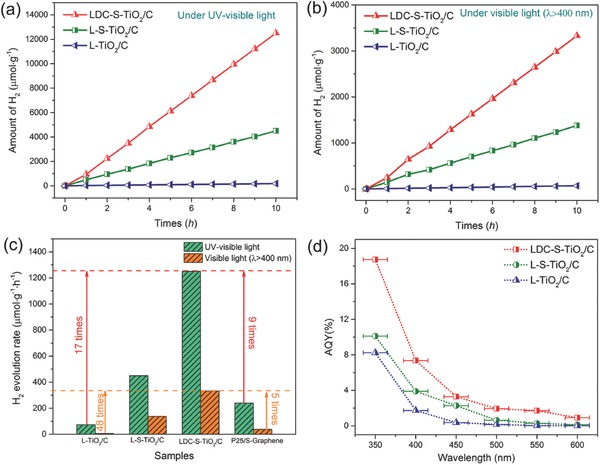
The comparisons of photocatalytic hydrogen generation rates of L‐TiO_2_/C, L‐S‐TiO_2_/C, and LDC‐S‐TiO_2_/C when using 0.1 g photocatalyst coated by 1% Pt cocatalyst in methanol–water solution under UV–vis light irradiation a) and under visible light irradiation b). c) Average photocatalytic hydrogen evolution rates of L‐TiO_2_/C, L‐S‐TiO_2_/C, LDC‐S‐TiO_2_/C, and P25/S‐Graphene with 1% Pt cocatalyst measured at atmospheric pressure. d) The apparent quantum yield (AQY) of L‐TiO_2_/C, L‐S‐TiO_2_/C, and LDC‐S‐TiO_2_/C with 1% Pt cocatalyst measured at atmospheric pressure.

Although doping often extends light absorption, photocatalytic activity is not always enhanced due to uncontrolled surface defects that work as recombination center. **Figure** **5**a shows the photocatalytic H_2_ generation performance during full‐band light irradiation for 10 h. After 10 h, L‐TiO_2_/C delivered very low H_2_ generation amount of 744 µmol g^−1^. Nevertheless, the H_2_ evolution rate of L‐TiO_2_/C is higher than that of bare rutile TiO_2_ (Figure S14, Supporting Information) owing to the high electrical conductivity of carbon substrate and fast charge separation induced by the junction architecture. With sulfur doping, L‐S‐TiO_2_/C delivered higher H_2_ amount of 4494 µmol g^−1^. The enhancement of H_2_ evolution amount of L‐S‐TiO_2_/C is attributed the broaden absorption wavelength. After air oxidation, the H_2_ generation of LDC‐S‐TiO_2_/C amounts to 12505 µmol g^−1^, likely owing to reduced defect concentration, enlarged SSA and the reduced excitation energy proved by modeling calculation later. This enhancement of approximately three times is not only attributed to the SSA. The catalytic profile of carbon layers is very important and should be responsible for high activity of LDC‐S‐TiO_2_/C as discussed later. Figure 5b shows the photocatalytic H_2_ evolution performance under visible light irradiation (λ ≥ 400 nm) during 10 h. The H_2_ evolution amount of L‐TiO_2_/C_,_ L‐S‐TiO_2_/C, and LDC‐S‐TiO_2_/C is 70, 1378, and 3330 µmol g^−1^, respectively. These results suggest that with S doping and air oxidation, the photocatalytic activity has been highly improved by a factor of nearly 50. To further reveal the cocatalyst' role of carbon substrate, we further compared the LDC‐S‐TiO_2_/C and L‐S‐TiO_2_/C photocatalyst with and without Pt cocatalysts in Figure S15 in the Supporting Information. One can see that the prepared carbon substrate can play similar role to Pt cocatalysts. Figure 5c shows the average H_2_ evolution rates calculated from Figure 5a,b. The H_2_ generation rate of LDC‐S‐TiO_2_/C under UV–vis light irradiation and visible light irradiation are 1250.5 and 333 µmol g^−1^ h^−1^, separately, which is 17 times and 48 times that of C/TiO_2_, and 5.2 times and 8.9 times that of the composites of commercial P25 and S‐doped Graphene. Finally, a full comparison with other reported TiO_2_ for photocatalytic hydrogen production is shown in Table S1 in the Supporting Information. The photocatalytic activity of the LDC‐S‐TiO_2_/C is superior compared with those previously reported TiO_2_‐based photocatalytic catalysts.^19^, ^34^ It is worth mentioning that the phase of TiO_2_ catalysts mentioned in Table S1 in the Supporting Information is mainly anatase TiO_2_, which is the most widely reported photocatalyst. The hydrogen evolution rate of 333 µmol g^−1^ h^−1^ is high compared with rutile photocatalyst because the reported rutile TiO_2_ suffers from low photocatalytic activity under visible light. Although rutile TiO_2_ take a large proportion in our synthesized materials, the LDC‐S‐TiO_2_/C delivered remarkable photocatalytic activity. The apparent quantum yield (AQY) of LDC‐S‐TiO_2_/C are measured (Figure 5d) to be 18.74% (at 350 nm), 7.36% (at 400 nm), 3.29% (at 450 nm), 1.94% (at 500 nm), and 1.71% (at 550 nm), respectively. In addition, an AQY of 0.92% measured at 600 nm suggests the TiO_2_‐based photocatalyst is active even at longer wavelengths, which is also consistent with the measured UV–vis absorption spectra.

**Figure 6:**
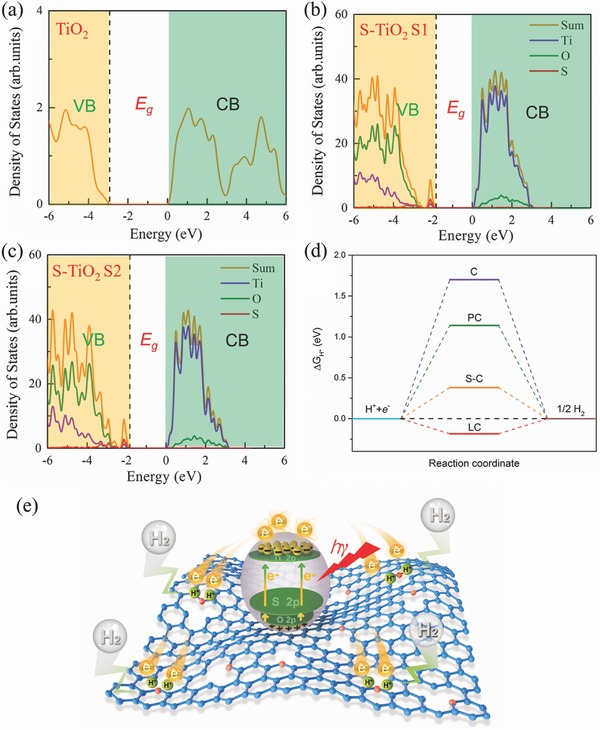
a) The calculated band structure of nondoped rutile TiO_2_. b,c) The calculated band structure of S‐TiO_2_ with one S atom at S1, and S2 doping sites in one 2 × 2 × 2 supercell, respectively. The dashed line denotes the Fermi level. d) Plots of Δ*G*_H*_ for different carbon‐based materials, where C represents for perfect graphene layer, PC represents for porous carbon, S‐C represents for sulfur‐doped carbon, and LC represents for laminated carbon with holes and sulfur doping. e) Mechanism of photocatalytic H_2_ generation of LDC‐S‐TiO_2_/C, in which the carbon atoms are marked in blue and the sulfur atoms are marked in red.

To further elucidate the enhanced activity of LDC‐S‐TiO_2_/C, the density functional theory (DFT) calculations were carried out. The S‐TiO_2_ photoabsorbers and laminated carbon (LC) cocatalysts were separately discussed. First, the effects of sulfur doping on the electronic structures of TiO_2_ were discussed. Considering that rutile TiO_2_ is the major component in the LDC‐S‐TiO_2_/C, the DFT calculations based on Heyd–Scuseria–Ernzerhof hybrid functional (HSE06) method were mainly based on the rutile TiO_2_. The energy band structures of rutile TiO_2_ are shown in **Figure** **6**a. A direct band gap of 2.97 eV was observed. A 2 × 2 × 2 supercell with one S atom doping was carried out to study the effects of S doping on the band structure. The structure of 2 × 2 × 2 supercell and two doping sites (S1 and S2) are shown in Figure S17 in the Supporting Information. The S doping content is around 3.1 at%, which is close to our experimental percentage of S (≈3.4 at%) by the elemental analyzer. An obvious valence band (VB) contribution from sulfur doping can be clearly observed at the top of the original VB. The band gaps for both S1 and S2 in the Supporting Information doping sites are reduced to 1.82 eV (Figure 6b,c). The shift of band gap indicating that S doping can significantly decrease the band gap, which is close to the experimental data. All calculated band gaps are slightly larger than that of our experimental values. This is owing to the existence of carbon in LDC‐S‐TiO_2_/C sample. Next we performed a series of DFT calculations for ∆*G*_H*_ to elucidate the function of carbon substrate. Theoretically, the HER can be described as a three‐state diagram containing (i) state of H^+^ + e^−^, (ii) state of adsorbed H (H*, * denotes an adsorption site), and (iii) state of 1/2 H_2_ product.[[qv: 17a]] Generally, a Gibbs free energy of adsorbed H atom approximating to zero (Δ*G*_H*_ ≈ 0) can provide a fast carrier‐transfer step and hydrogen molecule release process, leading to high catalytic activity.^35^ Because the reduction of H^+^ and the release of H_2_ mainly occur on carbon substrate, herein we only investigated the effects of DC on the hydrogen evolution activity. The optimal structure of H* adsorbed on carbon, porous carbon (PC), S‐doped carbon (S‐C), and LC (porous carbon with sulfur doping) are shown in Figure S18 in the Supporting Information. Figure 6d shows the calculated Δ*G*_H*_ for a series of carbon‐based materials. The single‐layer carbon, so‐called graphene, has an extremely high Δ*G*_H*_ of 1.70 eV, which is unfavorable for photocatalysis, consistent with the reported literatures.^17^ According to the computational results, it was considered that the porous hole and sulfur doping are two efficient approach to reduce the Δ*G*_H*_,[[qv: 19a,36]] resulting in a lower Δ*G*_H*_ of 1.14, 0.38 eV, respectively. Remarkably, owing to the synergistic effect of S‐doping and holes, the Δ*G*_H*_ value of LC is depressed to −0.185 eV, implying that laminated porous carbon substrate is a perfect cocatalyst for hydrogen production. The porous holes in carbon substrate can not only provide more active sites for photocatalysis but also accelerate proton reduction, which is well agreed with the EIS results.

Based on the discussion above, we conclude that the synergistic effects of S‐TiO_2_ with enhanced light harvesting and defective carbon cocatalytic effect are responsible for the improved photocatalytic activity. Figure 6e illustrates the whole photocatalytic H_2_ evolution processes. After S‐TiO_2_ absorbed the visible light, the electrons could be excited from the VB to the conduction band (CB) or from the sulfur dopant level to the CB, and photoexcited electrons–holes were generated. Then the photoexcited electrons could be quickly transferred to LC owing to the high conductivity of LC for electrons and close contact at interface. The holes will stay in TiO_2_. Thus, the electrons–holes were highly separated and the recombination was reduced. After that, owing to the LC's cocatalytic effect, the proton could be easily reduced and H_2_ molecules were produced. The roles of LC in photocatalysis can be summarized as follows: (i) reduce the charge transfer resistance and charge recombination, (ii) provide more active sites for photocatalysis, (iii) shorten the diffusion path of electrons, and (iv) more importantly accelerate proton reduction.

**Figure 7:**
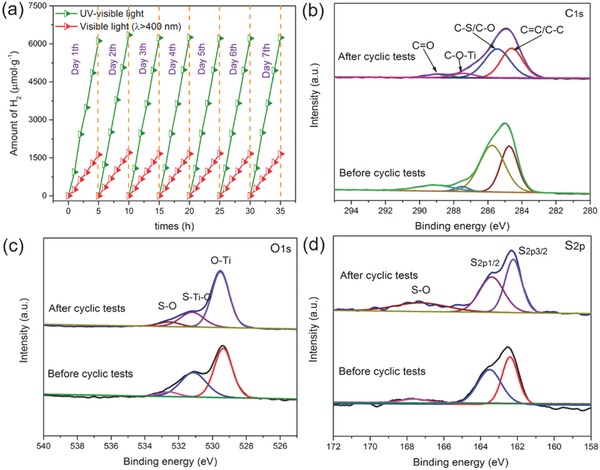
a) Stability test of the LDC‐S‐TiO_2_/C, XPS C1s b), XPS O1s c), and XPS S2p d) of LDC‐S‐TiO_2_/C before and after cyclic tests.

Photocatalytic stability is a key parameter for practical application, so the stability for the LDC‐S‐TiO_2_/C was tested by 5 h visible light irradiation/day in 7 d, as shown in **Figure** **7**a. The amount of produced H_2_ increases linearly with irradiation time. After 7 d, the photocatalytic activity still has been well preserved. The calculated activity retention is 102%, suggesting an excellent stability. The high stability was also proved by the XRD patterns (Figure S19, Supporting Information) and XPS spectra (Figure 7b–d). The peak intensities of C—S, S—Ti—O, and S—O bonds do not change, suggesting that the structure of LDC‐S‐TiO_2_/C can be well preserved after longtime cycles.

## 3 Conclusion

In conclusion, we demonstrated a novel approach for the synthesis of laminated defect controlled S‐doped TiO_2_ on carbon substrate (LDC‐S‐TiO_2_/C) involving an S impregnation of Ti_3_C_2_ *MXenes* and the subsequent two‐step oxidation processes. This novel method can simultaneously achieve the doping of TiO_2_ and the defect‐engineered carbon substrate. The H_2_ evolution rate under visible light irradiation can reach up to 333 µmol g^−1^ h^−1^, in addition, a high AQY of 7.36% at 400 nm can be realized, owing to the synergistic effect of porous carbon substrate and S doping. Specially, the porous carbon substrate provides the pathway for electrons separation, leading to high charge separation efficiency with a large surface area. More importantly, laminated carbon substrate as a cocatalyst can significantly reduce the ∆*G*_H*_, provide more active sites, and shorten the diffusion path of electrons, which accelerates the photocatalytic hydrogen production. In parallel, sulfur doping can reduce the band gap of TiO_2_, leading to an outstanding response from UV to visible light. The hybrid is also composed of earth abundant elements, thus expecting as a low cost and efficient photocatalyst. All these together shed a new insight in material design strategy for highly active laminated hybrid photocatalysts for solar energy conversion and environmental purification.

## 4 Experimental Section

*The Preparation of Ti_3_C_2_*: Typically, 1 g Ti_3_AlC_2_ was added into 10 mL hydrofluoric acid (HF) (40 wt%). The solution was stirred for 48 h at 45 °C. After HF etching, the Ti_3_C_2_ was gained by centrifugation and washing with deionized water until pH ≈ 7. After filtration, the Ti_3_C_2_ powder was vacuum dried at 60 °C for 12 h.

*The Exfoliated Ti_3_C_2_*: 0.5 g obtained Ti_3_C_2_ was added into 5 mL NH_3_·H_2_O (25–28 wt%). The solution was stirred for 24 h at room temperature. After that, the solution was ultrasonically treated for 2 h in 200 W ultrasonic bath. The exfoliated Ti_3_C_2_ was gained by centrifugation and deionized water washing until pH ≈ 7. After that, the solution was vacuum dried at 50 °C for 12 h.

*The Preparation of L‐TiO_2_/C*: The exfoliated Ti_3_C_2_ was putted into a quartz tube furnace at 700 °C at 10 °C s^−1^ for 2 h with 150 sccm flowing CO_2_ gas. After naturally cooled down to room temperature, L‐TiO_2_/C was obtained. The LDC‐TiO_2_/C was obtained via air oxidation of L‐TiO_2_/C at 450 °C for 2 h.

*The Preparation of L‐S‐TiO_2_/C and LDC‐S‐TiO_2_/C*: Typically, 0.1 g exfoliated Ti_3_C_2_ was mixed with 0.18 g sulfur with ball mixing. After that, the Ti_3_C_2_/S mixture was transferred into 155 °C 100 mL Teflon lined stainless‐steel autoclave for 12 h. After cooling down to room temperature, the obtained powder was transferred into a quartz tube furnace, following by the calcination at 700 °C for 2 h in 150 sccm flowing CO_2_ gas. The heating rate is 10 °C s^−1^. After naturally cooling down, samples were denoted L‐S‐TiO_2_/C. The LDC‐S‐TiO_2_/C was obtained by further oxidation under air atmosphere. The air oxidation of L‐S‐TiO_2_/C was carried out at 450 °C furnace for 2 h. L‐S‐TiO_2_ without carbon was synthesized via air oxidation of L‐S‐TiO2/C under 450 °C for 4 h to burn carbon off.

*The Preparation of P25/S‐Graphene*: 0.05 g graphene oxides (GO) was mixed with 0.09 g sulfur with ball mixing. After that, the GO/S mixture was transferred into 155 °C 100 mL Teflon lined stainless‐steel autoclave for 12 h. After cooling down to room temperature, the obtained powder was transferred into a quartz tube furnace, following by the calcination at 700 °C for 2 h in ambient Ar atmosphere. The heating rate is 10 °C s^−1^. After cooled to room temperature, S‐Graphene was obtained. The P25/S‐Graphene was prepared by mixing commercial P25 (Shanghai Haiyi Scientific and Trading Co., Ltd, 97.5 wt%) with S‐Graphene (2.5 wt%) together.

*Characterizations*: Bruker D8 ADVANCE X‐ray diffractometer equipped with Cu *K*_ɑ_ radiation was used to obtain the XRD patterns. The morphology and structure were observed by scanning electron microscopy (S4700, Hitachi, Japan) equipped with an EDX spectrometer and TEM (Talos F200X, FEI, USA). The elemental composition and chemical bonds were tested via XPS (PHI 5400, PE, USA). The percentage of sulfur is further measured by an Elemental Analyzer (EA S‐5000, Analytik Jena AG, Germany). Raman spectra were obtained by Renishaw Ramascope (Confocal Raman Microscope, Renishaw, Gloucester‐shire, UK) equipped with an He–Ne laser (λ = 532 nm). The specific surface area and pore size distribution were tested using the BET method (ASAP 2020, Micromeritics). A UV–vis spectrophotometer (UV‐3100, Shimadzu) was applied to obtain the UV–vis spectra at room temperature. The EPR spectra were tested using a digital X‐band spectrometer (EMX‐220, Bruker, Billerica, MA, USA) under 77 K. The EIS measurement was carried out on an electrochemical workstation (CHI‐660E, China) in a standard three‐electrode system. A Pt wire serviced as a counter electrode, a saturated Ag/AgCl electrode serviced as the reference electrode, and indium tin oxide (ITO) was working electrode. The Nyquist plots were recorded from 100 MHz to 100 kHz frequency range in a 0.1 m KCl solution containing 5 × 10^−3^ m Fe(CN)_6_^3−^/Fe(CN)_6_^4−^ as electrolyte solutions, respectively. A 500 W Xenon lamp with 400 nm UV cut‐off filter was utilized as the photosource.

*The Photoactivity Hydrogen Evolution Performance*: Water splitting was carried out in a lateral irradiation reaction vessel. A 300 W Xenon lamp (MAX‐302; Asahi Spectra, Torrance, CA, USA) without or with 400 nm UV cut‐off filter was used as the UV–vis or visible light irradiation source. 100 mg of photocatalyst powder was dispersed in 100 mL of aqueous solution containing 10 mL of methanol in volume (10 vol%) as the sacrificial agent for H_2_ evolution test. The deposition of 1% Pt cocatalysts was conducted by injecting 500 µL of 0.2 g L^−1^ H_2_PtCl_6_ solution into the above solution. The reaction temperature was kept at room temperature. The amount of produced H_2_ was determined by a gas chromatograph. The nitrogen was utilized as the carrier gas. AQY was calculated by using the following formula

(1) $$\mathrm{AQY}=\frac{\left(2\times \mathrm{the}\;\mathrm{number}\;\mathrm{of}\;\mathrm{evolved}\;\mathrm{hydrogen}\;\mathrm{molecules}\right)}{\mathrm{the}\;\mathrm{number}\;\mathrm{of}\;\mathrm{incident}\;\mathrm{photons}}\times 100\%$$

The light intensity was measured by an optical power meter (PD 130, Perfect Light, China) with an appropriate band‐pass filter (350, 400, 450, 500, 550, 600 nm, λ ± 15 nm at 10% of peak height) inserted between the 300 W Xe light source and the reactor.

*DFT Calculations*: The first‐principle calculations were performed by adopting the DFT methods implemented in the Vienna ab initio simulation package. The projector augmented wave pseudopotentials were employed to describe the interactions between valence electrons and ionic cores. The Perdew–Burke–Ernzerhof form of the generalized gradient approximation was adopted to describe electron exchange and correlation. The HSE06^36^ was employed to investigate the electronic properties of pristine TiO_2_ and S‐doped TiO_2_, respectively. The S‐TiO_2_ system was modeled by using single S atom doping in a 2 × 2 × 2 rutile TiO_2_ supercell. A gamma centered 5 × 5 × 8 *k*‐point mesh was employed to sample the irreducible Brillouin zone for all the calculations. The energy cutoff was set to 500 eV. The lattice constants and all atoms were fully relaxed until the maximum force on a single atom was smaller than 0.02 eV Å^−1^ by using conjugate gradient algorithm. The model of carbon is constructed as 7 × 7 periodic supercell comprising 98 C atoms. The Gibbs free energy change of H atoms bound to catalysts of the HER is calculated by the free energy with respect to molecular hydrogen including zero‐point energy and entropy terms, expressed as

(2) $$\Delta G_{H}=\Delta E_{H}+\Delta E_{\mathrm{ZPE}}-T\Delta S_{H}$$

where Δ*E*_H_, Δ*E*_ZPE_, and Δ*S*_H_ are the adsorption energy of hydrogen, the difference in zero point energy between the adsorbed hydrogen and hydrogen in gas phase, and the entropy difference between adsorbed state and gas phase. The entropy of atomic hydrogen can be taken as Δ*S*_H_ = − *S*_H2_/2, where *S*_H2_ is the entropy of molecule hydrogen in gas phase. In standard conditions Δ*E*_ZPE_ − *T*Δ*S*_H_ is about 0.24 eV, simplifying Equation (2) to Δ*G*_H_ = Δ*E*_H_ + 0.24.

## Conflict of Interest

The authors declare no conflict of interest.

## Supporting information

Supplementary
