# Supplementary material for: Laminated Hybrid Junction of Sulfur‐Doped TiO2 and a Carbon Substrate Derived from Ti3C2 MXenes: Toward Highly Visible Light‐Driven Photocatalytic Hydrogen Evolution
Source: Adv Sci (Weinh). 2018 Mar 30;5(6):1700870. doi: 10.1002/advs.201700870 (PMC6010755; doi:10.1002/advs.201700870)
Supplement: Supplementary file 1 — Supplementary [file ADVS-5-1700870-s001.pdf]

Copyright WILEY-VCH Verlag GmbH & Co. KGaA, 69469 Weinheim, Germany, 2018.

ADVANCED SCIENCE
Open Access

Supporting Information

for *Adv. Sci.,* DOI: 10.1002/advs.201700870

Laminated Hybrid Junction of Sulfur-Doped TiO_2 and a Carbon Substrate Derived from Ti_3C_2 MXenes: Toward Highly Visible Light-Driven Photocatalytic Hydrogen Evolution

Wenyu Yuan, Laifei Cheng, Yurong An, Shilin Lv, Heng Wu, Xiaoli Fan, Yani Zhang, Xiaohui Guo,* and Junwang Tang*

Copyright WILEY-VCH Verlag GmbH & Co. KGaA, 69469 Weinheim, Germany, 2016.

Supporting Information

Laminated Hybrid Junction of Sulfur-doped TiO_2 and Carbon Substrate Derived from Ti_3C_2 MXenes: towards Highly Visible Light -driven Photocatalytic Hydrogen Evolution

Wenyu Yuan, Laifei Cheng, Yurong An, Shilin Lv, Heng Wu, Xiaoli Fan, Yani Zhang, Xiaohui Guo,* Junwang Tang*

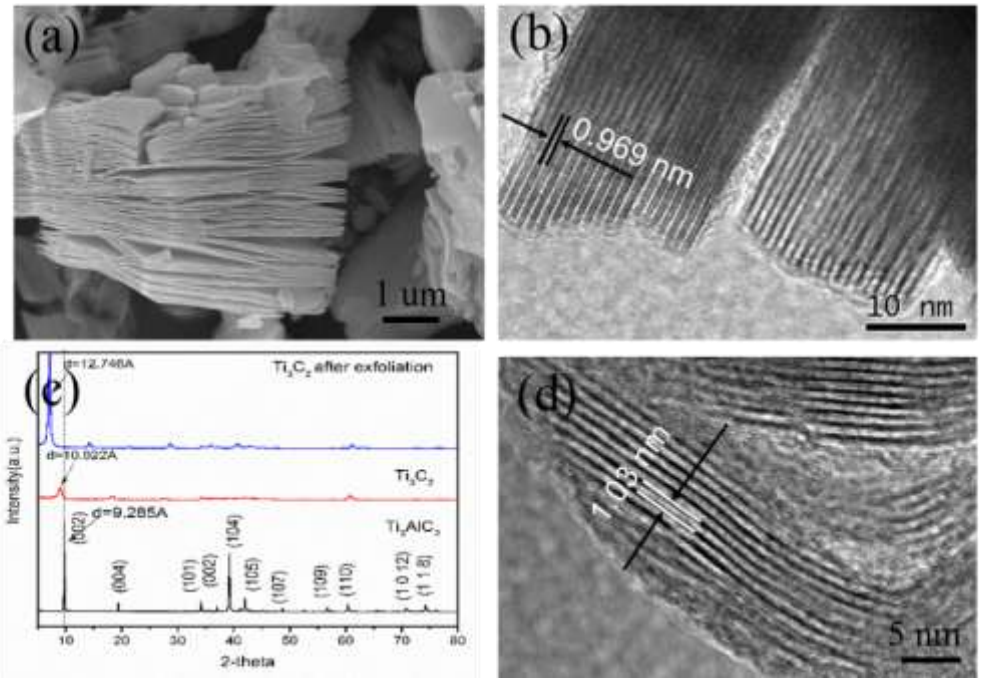

Figure S1. (a) SEM of Ti_3C_2. (b) HRTEM of Ti_3C_2. (c) XRD patterns of Ti_3AlC_2, Ti_3C_2 and exfoliated Ti_3C_2. (d) HRTEM of exfoliated Ti_3C_2

After HF etching, the (002) peak of Ti_3AlC_2 was shifted to lower 2-theta degree and has been broaden and weaken, suggesting that Ti_3C_2 was successfully prepared. The morphology of Ti_3C_2 are shown in Figure S1a. The interplanar spacing of (002) measured in Figure S1b is 0.969 nm. After exfoliation, the (002) peak was shifted to ~7.0^o, indicating that the d-spacing has been enlarged after exfoliation, which is in line with the HRTEM results in **Figure S1d**. the d-spacing measured in **Figure S1d** was 1.03 nm.

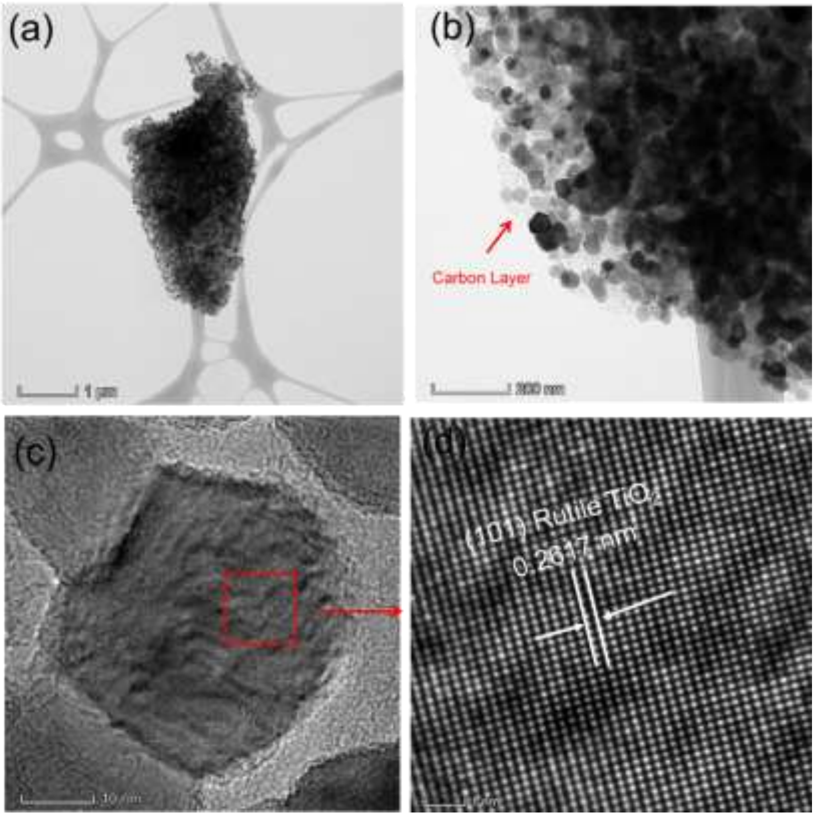

Figure S2. (a)The TEM images of L-S-TiO_2/C. (b-d) The HRTEM of L-S-TiO_2/C.

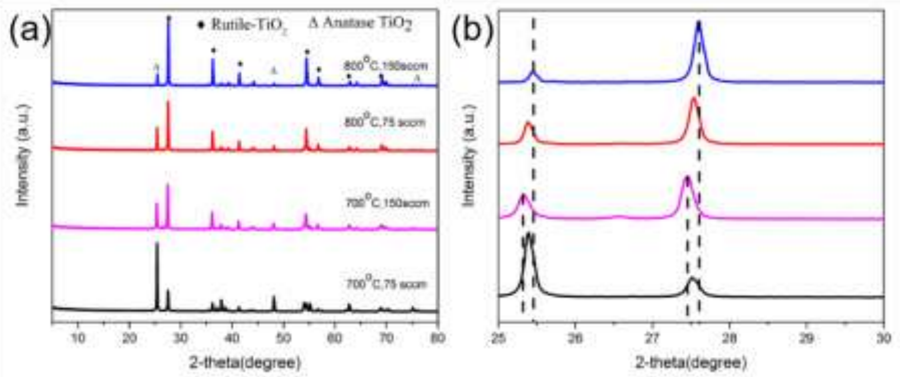

Figure S3. (a) XRD patterns of LDC-S-TiO_2/C obtained from different temperature; and (b) enlarged peaks between 25-30 ^{o} of LDC-S-TiO_2/C.

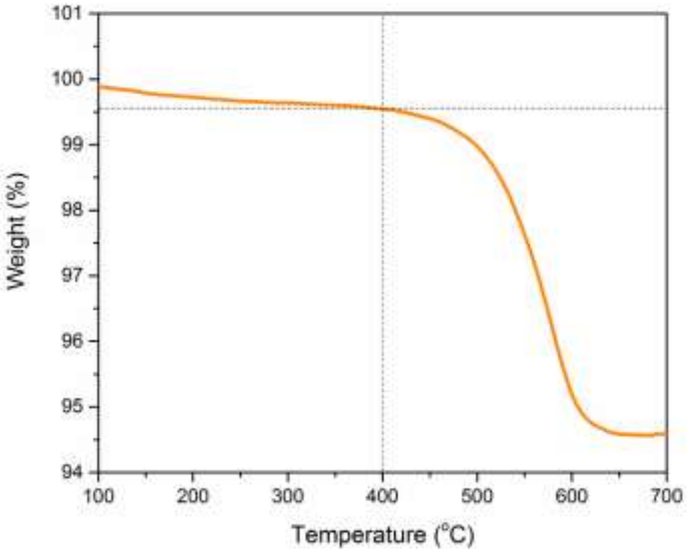

Figure S4. TG curves of L-S-TiO_2/C in air atmosphere.

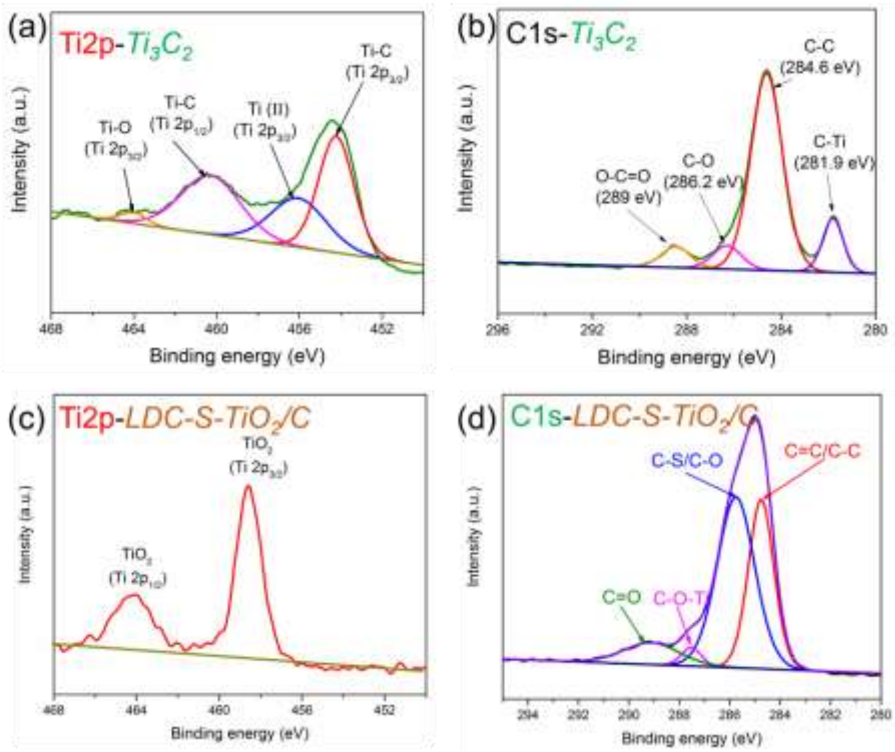

Figure S5. Ti2p and C1s XPS spectra of Ti_3C_2 (a, b) and LDC-S-TiO_2/C (c, d)

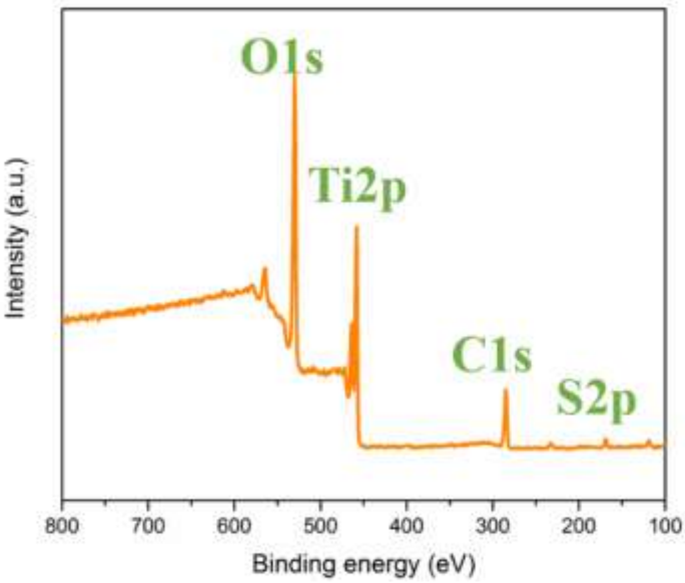

Figure S6. XPS survery of LDC-S-TiO_2/C

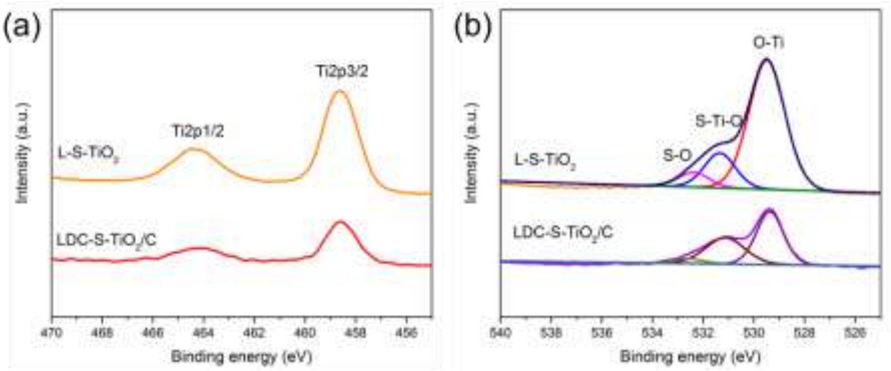

Figure S7. Ti2p XPS (b), and C1s (d) spectra of Ti_3C_2 and LDC-S-TiO_2/C.

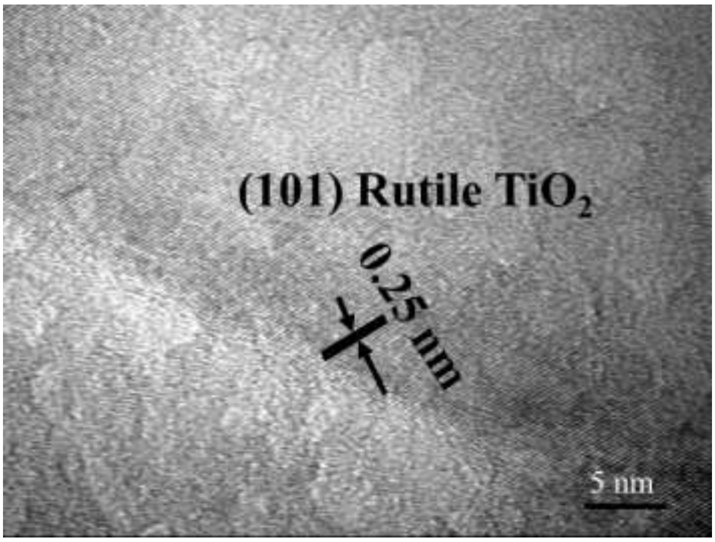

Figure S8. HRTEM of non-doped rutile TiO_2.

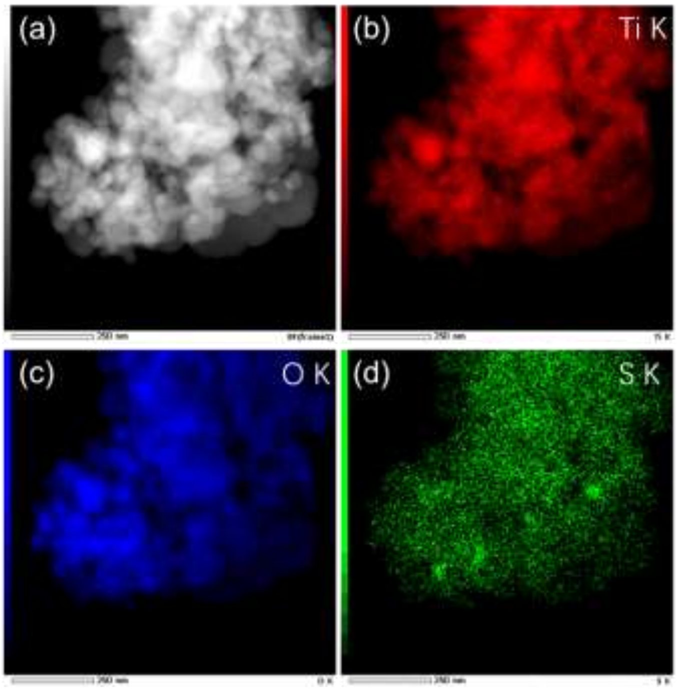

Figure S9. (a) the image of L-S-TiO_2 without carbon for EDX mapping. (b-d) EDX mapping of Ti, O, and S elements.

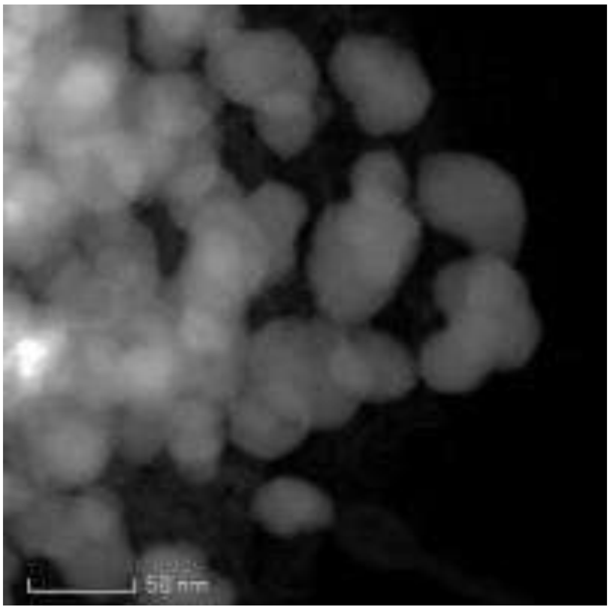

Figure S10. The image of LDC-S-TiO_2/C for EDX mapping.

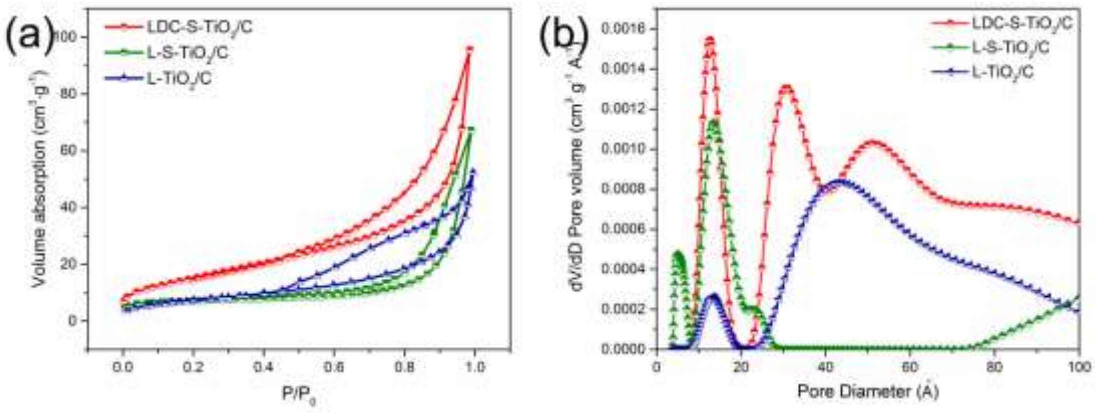

Figure S11. (a) N_2 adsorption/desorption isotherms and (b) the corresponding PSD of L-TiO_2/C, L-S-TiO_2/C, LDC-S-TiO_2/C.

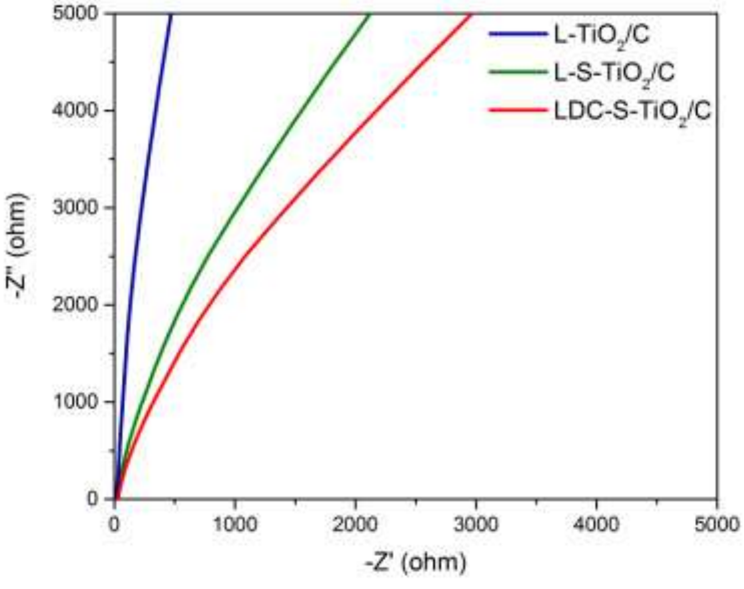

Figure S12. The Nyquist plot of L-TiO_2/C, L-S-TiO_2/C and LDC-S-TiO_2/C.

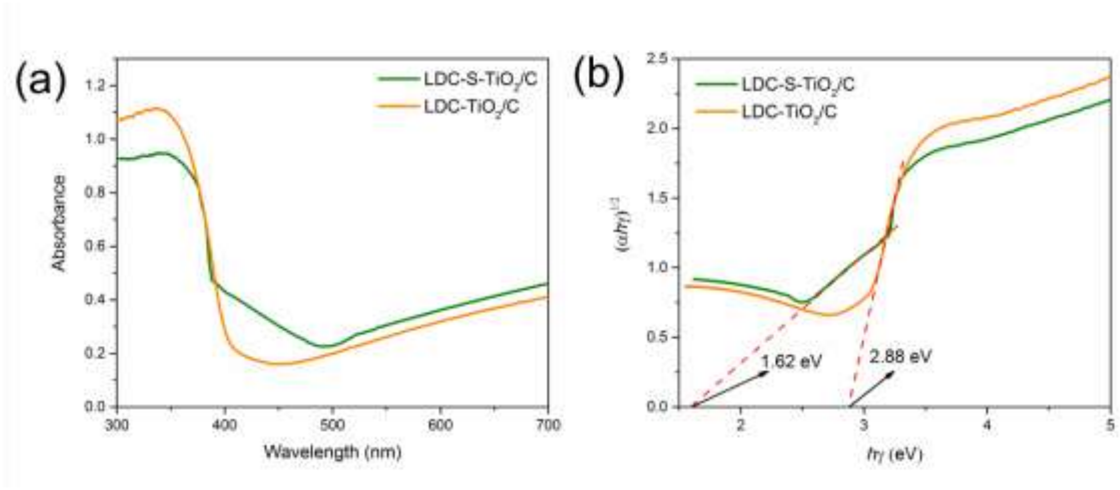

Figure S13. (a) UV-vis spectra and (b) $\alpha h\gamma^{1/2}$ versus photon-energy plot of LDC-TiO_2/C and LDC-S-TiO_2/C. The UV-vis spectra of L-S-TiO_2/C and L-TiO_2/C are not included because excess carbon make the absorbance higher than 1 and the band-structure unclear.

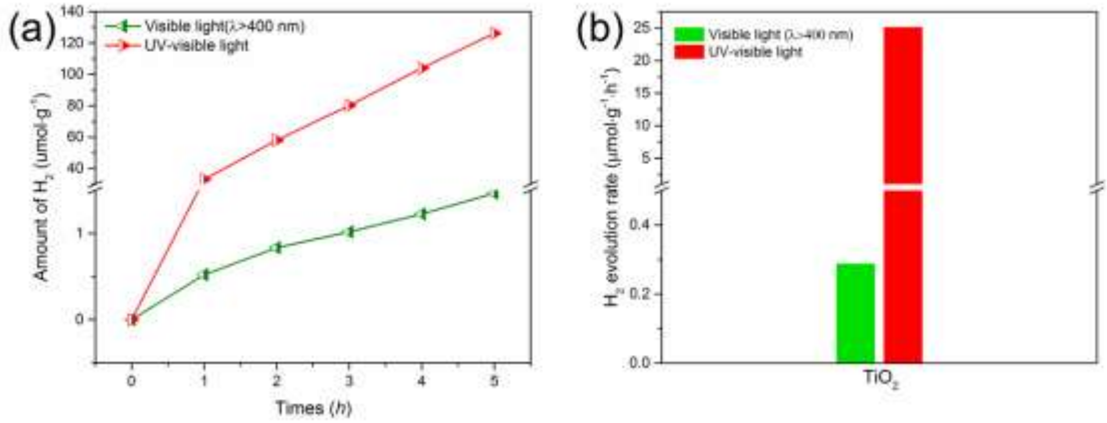

Figure S14. (a) The photocatalytic hydrogen generation rates and (b) the average photocatalytic hydrogen generation rates of bare rutile TiO_2 under UV-visible light irradiation and visible light irradiation using 0.1g photocatalyst coated by 1% Pt cocatalyst in methanol-water solution.

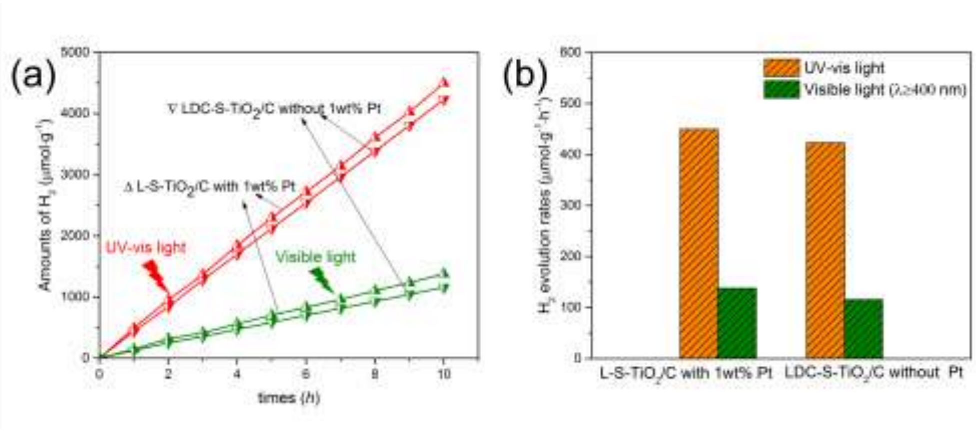

Figure S15. the H_2 evolution performance (a) and the H_2 evolution rates (b) of L-S-TiO_2/C and LDC-S-TiO_2/C in the presence or absence of 1wt% Pt as co-catalyst under UV-vis light and visible light irradiation.

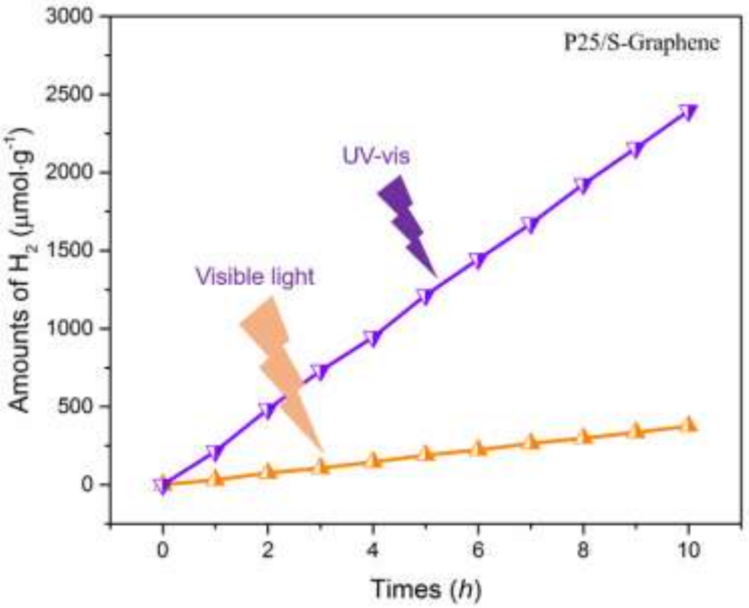

Figure S16. The H_2 evolution performance of the composites of commerical P25 and S-Graphene with 1wt% Pt as co-catalysts under UV-vis light and visible light irradiation. The hydrogen evolution rates of P25/S-Graphene are 37.6 $\mu mol \cdot g^{-1} \cdot h^{-1}$, 240 $\mu mol \cdot g^{-1} \cdot h^{-1}$ under visible light and UV-vis light irradiation.

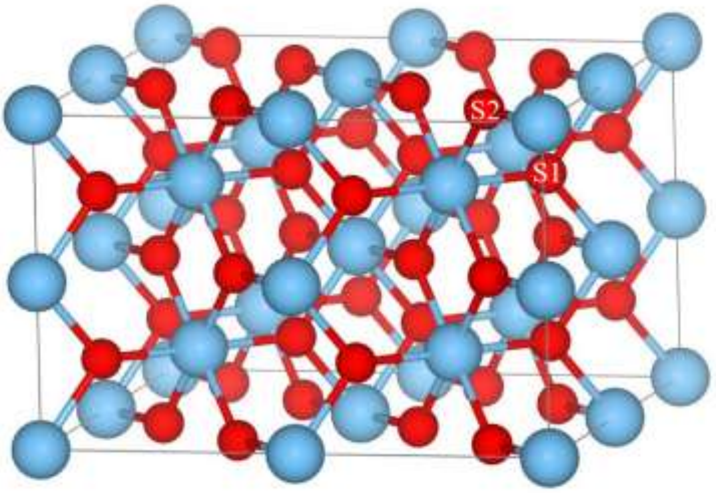

Figure S17. (a) the structure model for a 2×2×2 rutile TiO_2 supercell. The red and blue spheres denote O and Ti atoms, respectively. S1 and S2, which located at the sites in plane and inside of unit cell, repectively, means two different doping sites in rutile TiO_2.

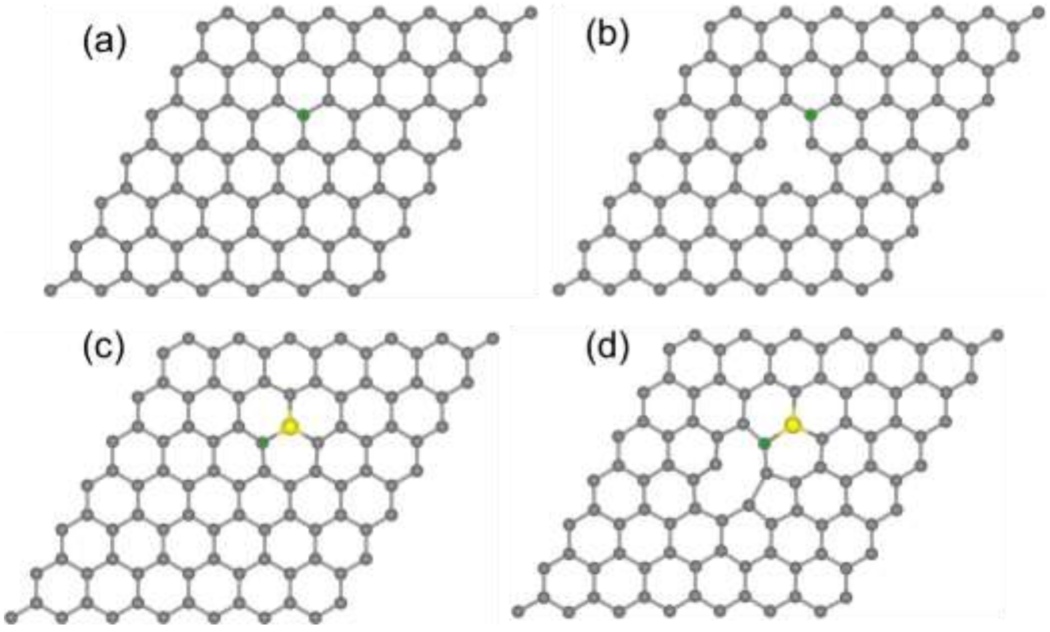

Figure S18. Optimized structure of H* adsorbed on carbon (a), PC (b), S-C (c) and LC with holes and S doping (d), in which the absorption sites are marked in blue and the sulfur atoms are marked in yellow.

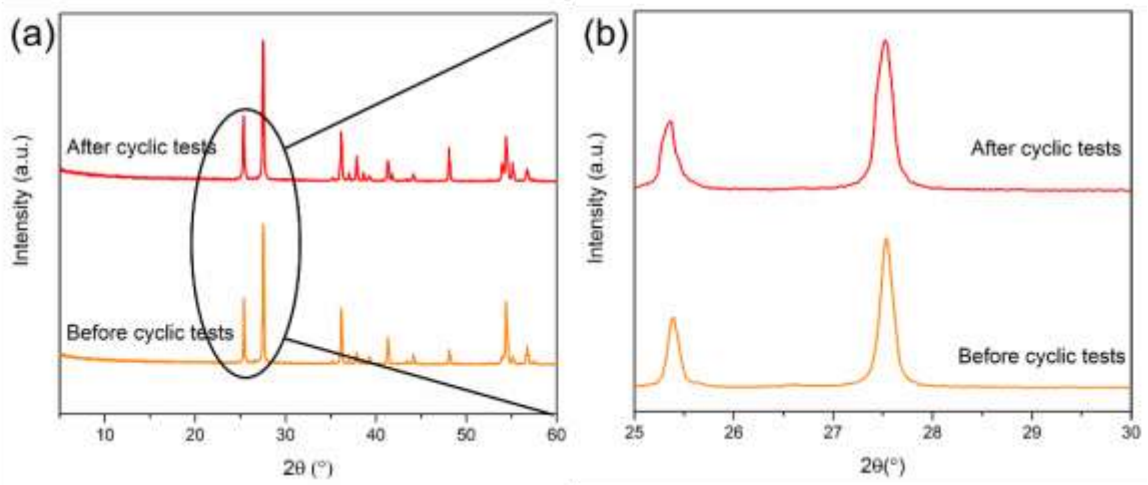

Figure S19. the XRD patterns (a) and the enlarged 25-30 ° XRD patterns of LDC-S-TiO_2/C before and after cyclic tests.

Table S1. Hydrogen evolution of LDC-S-TiO_2/C and comparison with other reported TiO_2 catalysts

Materials	Co-catalyst	Light (nm)	Sacrificial reagent	Activity ($\mu mol \cdot g^{-1} \cdot h^{-1}$)	References
LDC-S-TiO_2/C	Pt	>400	Methanol	333	This work
Al-reduced TiO_2	Pt	>400	Methanol	140	1
Black TiO_2 hollow spheres	Pt	>365	Methanol	241	2
TiO_2–In_2O_3/g-C_3N_4	None	>400	Methanol	160	3
Graphene-Au-TiO_2	Au	>420	Methanol	296	4
S,N-modified TiO_2	Au	>400	Methanol	267.6	5
Black TiO_2	Pt	>400	Methanol	100	6

References

[1] Z. Wang, C. Yang, T. Lin, H. Yin, P. Chen, D. Wan, F. Xu, F. Huang, J. Lin, X. Xie, *Energy & Environ. Sci.* **2013**, *6*, 3007.

[2] W. Hu, W. Zhou, K. Zhang, X. Zhang, L. Wang, B. Jiang, G. Tian, D. Zhao, H. Fu, *J. Mater. Chem. A* **2016**, *4*, 7495.

[3] Z. Jiang, D. Jiang, Z. Yan, D. Liu, K. Qian, J. Xie, *Appl. Catal. B: Environ.* **2015**, *170*, 195.

[4] Y. Wang, J. Yu, W. Xiao, Q. Li, *J. Mater. Chem. A* **2014**, *2*, 3847.

[5] S. Pany, B. Naik, S. Martha, K. Parida, *ACS Appl. Mater. & Interfaces* **2014**, *6*, 839.

[6] X. Chen, L. Liu, Y. Y. Peter, S. S. Mao, *Science* **2011**, *331*, 746.
